# Socioeconomic position and risk of short-term weight gain: Prospective study of 14,619 middle-aged men and women

**DOI:** 10.1186/1471-2458-8-112

**Published:** 2008-04-09

**Authors:** Lisa R Purslow, Elizabeth H Young, Nicholas J Wareham, Nita Forouhi, Eric J Brunner, Robert N Luben, Ailsa A Welch, Kay-Tee Khaw, Shelia A Bingham, Manjinder S Sandhu

**Affiliations:** 1 MRC Epidemiology Unit, Institute of Metabolic Science, Cambridge, UK; 2 Cancer Research UK Health Behaviour Research Centre, UCL, London, UK; 3 Department of Public Health and Primary Care, University of Cambridge, Cambridge, UK; 4 Department of Epidemiology and Public Health, UCL, London, UK; 5 MRC Dunn Human Nutrition Unit, Cambridge, UK; 6 MRC Centre for Nutrition in Cancer Epidemiology Prevention and Survival, Department of Public Health and Primary Care, University of Cambridge, Cambridge, UK

## Abstract

**Background:**

The association between socioeconomic position in middle age and risk of subsequent, short-term weight gain is unknown. We therefore assessed this association in a prospective population based cohort study in Norfolk, UK.

**Methods:**

We analysed data on 14,619 middle-aged men and women (aged between 40–75 at baseline) with repeated objective measures of weight and height at baseline (1993–1997) and follow up (1998–2000).

**Results:**

During follow up 5,064 people gained more than 2.5 kg. Compared with the highest social class, individuals in the lowest social class had around a 30% greater risk of gaining more than 2.5 kg (OR 1.29; 95% CI 1.11–1.51; p for trend = 0.002). This association remained statistically significant following adjustment for sex, age, baseline BMI, smoking, and follow up time (OR 1.25; CI 1.07–1.46; p for trend <0.001). We also found no material difference between unadjusted models and those including all confounders and potential mediators.

**Conclusion:**

Individuals of low socioeconomic position are at greatest risk of gaining weight during middle age, which is not explained by classical correlates of socioeconomic position and risk factors for obesity.

## Background

Across the UK there has been a rapid increase in the prevalence of obesity in recent decades [1]. Since obesity is associated with a greater risk of morbidity and mortality [2], identifying the determinants of weight gain and obesity is fundamental to the development of preventative strategies at the individual and societal level. Social inequalities in health are well recognised, and several studies suggest that adverse socioeconomic position is associated with obesity [3-5]. However, the association between socioeconomic position in middle age and risk of subsequent, short-term weight gain is unknown [5]. Additionally, few studies have attempted to investigate the mechanisms underlying the associations between socioeconomic position and weight gain [5,6]. We therefore investigated this association in a prospective population based study of 14,619 middle-aged men and women.

## Methods

We used data from the European Prospective Investigation into Cancer and Nutrition (EPIC) Norfolk cohort. The study was approved by the Norfolk Health District Ethics Committee and full details of participant recruitment and study procedures have been published previously [7]. Briefly, recruitment started in March 1993 and was completed by the end of 1997 when 25,631 individuals (aged 40 – 75) had attended a baseline health check. Between 1998 and 2000 15,028 individuals completed a follow up health check. Of these participants we analysed data on 14,619 (97%) men and women who had complete data and did not report stroke, cancer or heart attack at baseline. Height and weight were measured and blood samples taken [8]. All participants completed a food frequency [9] and health and lifestyle questionnaire. Physical activity was assessed using a previously validated 4-level occupation and recreation activity index [10]. Physical activity and dietary data were only collected at baseline. We used social class to categorise socioeconomic position, based on the Registrar General's occupational classification: I professional; II intermediate; IIIa skilled non-manual; IIIb skilled manual; IV semi-skilled; and V unskilled [11,12].

All analyses were carried out using STATA version 8 (Stata Corporation, College Station, Texas). Linear regression analyses were used to assess the association between socioeconomic position, baseline BMI, and weight change over the follow up period. There was no material difference in baseline BMI (p = 0.73) or weight change over follow up (p = 0.23) between individuals with no social class coding and all other individuals.

Weight change is a complex phenomenon encompassing individuals who gain weight (positive energy balance), lose weight (negative energy balance) and remain weight stable (energy balanced) [13]. We therefore also assessed the association between socioeconomic position and weight gain using an arbitrary cut-off of 2.5 kg [13]. Specifically, we assessed the relation between socioeconomic position and positive energy balance by comparing individuals who gained more than 2.5 kg over the follow up period with those who maintained a stable weight or lost weight (≤ 2.5 kg) using logistic regression.

In subsequent analyses, we explored whether behaviours related to socioeconomic position (diet and physical activity) mediated these associations. We have previously shown that plasma vitamin C levels in this cohort positively correlate with fruit and vegetable intake [14]; we therefore used this biomarker as an indicative measure of fruit and vegetable intake.

## Results

Complete anthropometric data were available for 14,619 of the 15,028 (97.3%) individuals who attended both health checks. Compared with the highest social class, mean BMI was greatest in the lowest social class (Table 1) for both men (p for trend <0.001) and women (p < 0.001). As expected the proportion of smokers was greatest in the lowest social class (p for χ^2 ^test for heterogeneity <0.001 men, p < 0.005 women) and energy intake was highest in the lowest social class (p = 0.005 men, p < 0.001 women). At baseline there was a statistically significant interaction between sex and social class with BMI (p = 0.0001). Specifically, the positive association between social class and BMI was stronger in women.

**Table 1:** Characteristics of the EPIC-Norfolk cohort by social class measured at baseline from 1993 – 1997

	**I**	**II**	**IIIa**	**IIIb**	**IV and V**	P value*
**Men N**	526	2655	818	1459	965	
**Age (years)**	59.7 (0.40)	59.6 (0.18)	60.6 (0.31)	59.2 (0.23)	60.1 (0.27)	0.627
**Weight (kg)**	79.9 (0.46)	80.7 (0.21)	80.2 (0.37)	79.7 (0.28)	79.3 (0.37)	0.002
**Height (cm)**	175.5 (0.27)	175.0 (0.13)	174.4 (0.23)	173.3 (0.17)	172.7 (0.21)	<0.001
**BMI (kg/m**^2^**)**	25.9 (0.13)	26.3 (0.06)	26.3 (0.11)	26.5 (0.08)	26.6 (0.11)	<0.001
**Follow up time (years)**	3.6 (0.03)	3.7 (0.02)	3.6 (0.03)	3.7 (0.02)	3.6 (0.02)	0.084
						
**Smoking N (%)**^†^						
Current	24 (5)	196 (7)	72 (9)	175 (12)	134 (14)	<0.001^‡^
Former	255 (48)	1409 (53)	463 (57)	836 (57)	541 (56)	
Never	247 (47)	1050 (40)	283 (35)	448 (31)	290 (30)	
						
**Physical Activity N (%)**^†^						
Inactive	141 (27)	744 (28)	288 (35)	353 (24)	254 (26)	<0.001^‡^
Moderately inactive	190 (36)	786 (30)	237 (29)	224 (15)	168 (17)	
Moderately active	119 (23)	629 (24)	164 (20)	423 (29)	259 (27)	
Active	76 (14)	496 (19)	129 (16)	459 (31)	284 (29)	
**Energy Intake (kj)**^§^	9091 (111)	9162 (50)	9190 (87)	9476 (72)	9256 (87)	0.005
**Plasma Vitamin C (umol/l)**^#^	53.3 (0.86)	51.0 (0.36)	49.0 (0.69)	46.7 (0.51)	45.6 (0.64)	<0.001
**Women N**	574	3052	1619	1657	1294	
**Age (years)**	57.5 (0.37)	57.9 (0.16)	59.9 (0.23)	57.5 (0.21)	58.6 (0.24)	0.084
**Weight (kg)**	66.4 (0.47)	67.0 (0.20)	66.8 (0.27)	67.6 (0.28)	68.7 (0.34)	<0.001
**Height (cm)**	162.5 (0.25)	161.9 (0.11)	161.1 (0.15)	160.7 (0.15)	160.6 (0.17)	<0.001
**BMI (kg/m**^2^**)**	25.2 (0.17)	25.6 (0.07)	25.7 (0.10)	26.2 (0.10)	26.7 (0.13)	<0.001
**Follow up time (years)**	3.5 (0.03)	3.6 (0.01)	3.7 (0.02)	3.7 (0.02)	3.7 (0.02)	0.002
						
**Smoking N (%)**^†^						
Current	39 (7)	248 (8)	141 (9)	172 (10)	139 (11)	<0.005^‡^
Former	161 (28)	959 (31)	530 (33)	533 (32)	403 (31)	
Never	374 (65)	1845 (60)	948 (59)	952 (57)	752 (58)	
						
**Physical Activity N (%)**^†^						
Inactive	108 (19)	725 (24)	503 (31)	444 (27)	345 (27)	<0.001^‡^
Moderately inactive	197 (34)	1042 (34)	562 (35)	522 (32)	372 (29)	
Moderately active	171 (30)	778 (25)	341 (21)	380 (23)	301 (23)	
Active	98 (17)	507 (17)	213 (13)	311 (19)	276 (21)	
**Energy Intake (kj)**^§^	7982 (86)	8035 (40)	8143 (54)	8200 (57)	8288 (68)	<0.001
**Plasma Vitamin C (umol/l)**^#^	64.1 (0.88)	62.9 (0.39)	59.7 (0.49)	58.6 (0.48)	57.0 (0.58)	<0.001
						
**All N**	1100	5707	2437	3116	2259	
**Women N (%)**	574 (52)	3052 (53)	1619 (66)	1657 (53)	1294 (57)	0.008
**Age (years)**	58.6 (0.28)	58.7 (0.12)	60.2 (0.18)	58.3 (0.16)	59.3 (0.18)	0.165
**Weight (kg)**	72.9 (0.39)	73.4 (0.17)	71.3 (0.25)	73.3 (0.23)	73.3 (0.27)	0.913
**Height (cm)**	168.7 (0.27)	168.0 (0.12)	165.6 (0.18)	166.6 (0.16)	165.8 (0.18)	<0.001
**BMI (kg/m**^2^**)**	25.5 (0.11)	25.9 (0.05)	25.9 (0.07)	26.3 (0.07)	26.6 (0.08)	<0.001
**Follow up time (years)**	3.6 (0.02)	3.7 (0.01)	3.7 (0.01)	3.7 (0.01)	3.7 (0.02)	<0.001
						
**Smoking N (%)**^†^						
Current	63 (6)	444 (8)	213 (9)	347 (11)	273 (12)	<0.001^‡^
Former	416 (38)	2368 (41)	993 (41)	1369 (44)	944 (42)	
Never	621 (56)	2895 (51)	1231 (51)	1400 (45)	1042 (46)	
						
**Physical Activity N (%)**^†^						
Inactive	249 (23)	1469 (26)	791 (32)	797 (26)	599 (27)	<0.001^‡^
Moderately inactive	387 (35)	1828 (32)	799 (33)	746 (24)	540 (24)	
Moderately active	290 (26)	1407 (25)	505 (21)	803 (26)	560 (25)	
Active	174 (16)	1003 (18)	342 (14)	770 (25)	560 (25)	
						
**Energy Intake (kj)**^§^	8509 (71)	8561 (33)	8495 (47)	8797 (47)	8701 (55)	<0.001
**Plasma Vitamin C (umol/l)**^#^	58.8 (0.64)	57.3 (0.28)	56.1 (0.41)	53.0 (0.37)	52.1 (0.45)	<0.001

All values are means and standard errors unless otherwise stated.

* Social class is coded as 0, 1, 2, 3, 4 and treated as a continuous variable to calculate p for linear trend.

^† ^Some totals may exceed 100% due to rounding.

^‡ ^P value for χ^2 ^test for heterogeneity.

^§^N = 14,292

^# ^N = 13,042

For the prospective analyses, sex stratified results (see Additional file 1) were similar to combined results (men and women) for weight change and for weight gain (all p for interactions >0.3); we therefore present data for the combined analyses. Although baseline BMI was significantly negatively correlated with weight change (r = -0.05, p < 0.001) there was no statistically significant interaction between baseline BMI and social class with weight gain. Mean weight change was +1.42 kg (SE 0.08) for individuals in the lowest social class, whereas mean weight change was +0.97 kg (0.12) for those in the highest social class (p for trend = 0.016) (Table 2), reflecting an absolute mean difference of 0.45 kg (p = 0.002). These associations remained statistically significant after adjustment for sex, age, baseline BMI, smoking, and follow up time (p for trend <0.001). The average follow up time for the whole study population was 3.66 years (SE 0.01).

**Table 2:** Association between social class, baseline BMI and weight change from baseline to follow up of 6,423 men and 8,196 women in the EPIC-Norfolk cohort

**Social Class**	**Baseline BMI**		**Weight change over follow up (kg)**	
	**Unadjusted**	Adjusted*	**Unadjusted**	**Adjusted**^†^

**I**	25.5 (0.11)	25.5 (0.11)	0.97 (0.12)	0.94 (0.12)
**II**	25.9 (0.05)	25.9 (0.05)	1.30 (0.05)	1.28 (0.05)
**IIIa**	25.9 (0.08)	25.9 (0.07)	1.29 (0.08)	1.36 (0.08)
**IIIb**	26.3 (0.07)	26.4 (0.07)	1.36 (0.07)	1.34 (0.07)
**IV and V**	26.6 (0.08)	26.6 (0.08)	1.42 (0.08)	1.46 (0.08)
**P for trend**	<0.001	<0.001	0.016	<0.001

All values are means and standard errors unless otherwise stated.

* Adjusted for sex, age and smoking.

^† ^Adjusted for sex, age, baseline BMI, smoking and follow up time

Over the follow up period 5,064 people gained more than 2.5 kg (Table 3). Compared with those in the highest social class, individuals in the lowest social class had a greater risk of gaining more than 2.5 kg (OR 1.29; 95% CI 1.11–1.51; p for trend = 0.002). This association remained statistically significant following adjustment for sex, age, baseline BMI, smoking, and follow up time (OR 1.25; CI 1.07–1.46; p for trend <0.001).

**Table 3:** Association between social class and risk of gaining more than 2.5 kg over the follow up period of 6,423 men and 8,196 women in the EPIC-Norfolk cohort

**Social Class**	**Weight stable gain ≤ 2.5 kg N (%)**	**Weight gain >2.5 kg N (%)**	**Unadjusted odds ratio (95% CI)**	Adjusted odds ratio* **(95% CI)**
**I**	760 (8)	340 (7)	1	1
**II**	3760 (39)	1947 (38)	1.16 (1.01 1.33)	1.13 (0.98 1.30)
IIIa	1590 (17)	847 (17)	1.19 (1.02 1.39)	1.21 (1.04 1.41)
**IIIb**	2014 (21)	1102 (22)	1.22 (1.06 1.42)	1.16 (1.00 1.34)
**IV and V**	1431 (15)	828 (16)	1.29 (1.11 1.51)	1.25 (1.07 1.46)
**P for trend**			0.002	<0.001

* Adjusted for sex, age, baseline BMI, smoking and follow up time

To assess whether certain lifestyle factors mediated the association between socioeconomic position and weight change or gain, we included plasma vitamin C level, total energy intake and physical activity as covariates in the adjusted models (see Additional file 2). For weight change, based on a subset of 12,760 participants with complete data on confounders and potential mediators, there was no material difference between the unadjusted model and a model including all covariates (data not shown). Similarly, for weight gain there was no material difference between the unadjusted model (lowest social class compared to highest OR 1.35; CI 1.14–1.60; p for trend <0.001) and a model including all confounders and potential mediators (OR 1.33; CI 1.12–1.57; p for trend <0.001). Plasma vitamin C levels in this cohort positively correlate with fruit and vegetable intake [14] Using vitamin C as a correlate of dietary intake, we found that this biomarker was not associated with subsequent weight gain (data not shown).

## Discussion

Our data indicate that men and women of low socioeconomic position are more likely to gain weight in middle age than individuals of high socioeconomic position. Specifically, we found that individuals in the lowest category gained 0.45 kg more than those in the highest, over an average follow up time of 3.66 years. Similarly, compared with the highest social class, individuals in the lowest social class had around a 30% greater risk of gaining more than 2.5 kg over the follow-up period. These associations were not materially altered by adjustment for confounders or potential mediators, including baseline correlates of energy intake and expenditure. Our findings indicate that the mechanisms underlying these associations are complex, and are not explained by classical correlates of social inequality and obesity risk factors.

Prospective studies have reported on the association between socioeconomic position and weight change or gain in adulthood [6,15,16]. However, these results were based on self reported past and present weight. Because greater underreporting of weight occurs in overweight or obese individuals, and prevalence of obesity is greater in people with a low socioeconomic position, reliance on self-reported weight could lead to an underestimation of the true association between socioeconomic position and weight gain [17]. As far as we are aware, our study is the first to use objectively measured weight to show that socioeconomic position predicts short-term weight gain in middle-aged men and women.

Diet [18,19] and physical activity [20] are correlates of socioeconomic position, which may mediate the association between socioeconomic position and weight gain. Therefore we included plasma vitamin C level (as a marker of fruit and vegetable intake), energy intake, and physical activity, all measured at baseline, as potential mediators. Previous research has suggested that while behavioural factors such as diet and physical activity are associated with BMI, social gradients in these factors do not wholly explain socioeconomic differences in BMI [6,21]. In keeping with these findings, our results suggest that neither baseline, plasma vitamin C, energy intake nor physical activity is likely to fully account for the association between socioeconomic position and short-term weight gain. However, we cannot rule out the possibility that differential reporting of diet and physical activity by social class may have occurred.

As a limitation, we cannot exclude the possibility that random measurement error may explain why we found no evidence of mediation by diet or physical activity. In the context of systematic error, differential reporting of dietary intake and physical activity by weight gain status could distort the interrelation among socioeconomic position, potential mediators and weight gain. However, in the prospective analysis mediators were assessed prior to weight gain. Additionally we included plasma vitamin C level as an objective measure of dietary intake [14], which is an unbiased assessment. In addition it is worth noting that short-term weight gain is an ongoing process during adult life; it is therefore likely that some weight gain preceded the baseline measures and influenced behaviour prior to exposure measurement. We only examined the impact of baseline correlates of energy intake and expenditure on the association between socioeconomic position and weight gain. Changes in these correlates (for example, diets and physical activity) between baseline and follow up might also have an important impact on the magnitude of the association between socioeconomic position and weight gain. Changes in these factors during follow up might therefore mediate our observed association between baseline socioeconomic position and subsequent weight gain. Although the use of logistic regression to calculate the odds ratio may substantially overestimate the risk ratio, as a measure of association the odds ratio still has utility. Our aim was to examine whether there was an association between socioeconomic position and subsequent risk of obesity, and whether this was mediated by correlates of socioeconomic position. In order to confirm the magnitude of association between socioeconomic position and weight gain further research is needed.

## Conclusion

Our study indicates that the mechanisms underlying the association between social inequalities and weight gain are complex. Given our findings, it remains unclear which components or correlates of low socioeconomic position underlie its relation with short-term weight gain. Differences in dietary patterns, including fast food intake – which is associated with weight gain independently of education, smoking, physical activity and dietary intake of fruit and vegetables [22] – may explain this relation. Alternatively, psychological stress is associated both with low socioeconomic position and with eating behaviour, metabolism and fat distribution [21,23-26], and is a predictor of both general and central obesity [27]. Therefore, stress could mediate the socioeconomic position and weight gain association.

In conclusion, in this prospective analysis of 14,619 individuals, we have shown that individuals of low socioeconomic position are at greatest risk of gaining weight during middle age, which is not explained by baseline classical correlates of socioeconomic position and risk factors for obesity. In order to reliably confirm the magnitude of the association between social class and weight gain, additional studies contextualised with systematic overviews will be required.

## Competing interests

The author(s) declare that they have no competing interests.

## Authors' contributions

LRP and MSS devised the study. LRP obtained and analysed the data, and together with EHY and MSS interpreted the results and wrote the manuscript. All authors commented on earlier drafts and approved the final version of the manuscript.

## Pre-publication history

The pre-publication history for this paper can be accessed here:



## Supplementary Material

Additional file 1 Statistical analyses by sex. The data provided represent the statistical analysis stratified by sex. Click here for file

Additional file 2 Statistical analyses with full diet and physical activity data. The data provided represent the statistical analysis for only individuals with full diet and physical activity data available. Click here for file
