# Supplementary material for: Socioeconomic position and risk of short-term weight gain: Prospective study of 14,619 middle-aged men and women
Source: BMC Public Health. 2008 Apr 9;8:112. doi: 10.1186/1471-2458-8-112 (PMC2323377; doi:10.1186/1471-2458-8-112)
Supplement: Additional file 1 — Statistical analyses by sex. The data provided represent the statistical analysis stratified by sex. [file 1471-2458-8-112-S1.doc]

**Statistical analyses stratified by sex**

| **Table S1: Association between social class, baseline BMI, and weight change from baseline to follow up, for 6,423 men in the EPIC-Norfolk cohort** | | | | |
| --- | --- | --- | --- | --- |
| **Social Class** | **Baseline BMI** | | **Weight change over follow up (kg)** | |
|  | **Unadjusted** | **Adjusted*** | **Unadjusted** | **Adjusted**^†^ |
| I | 25.9 (0.13) | 25.8 (0.14) | 0.95 (0.16) | 0.97 (0.16) |
| **II** | 26.3 (0.06) | 26.3 (0.06) | 1.27 (0.07) | 1.29 (0.07) |
| **IIIa** | 26.3 (0.11) | 26.2 (0.11) | 1.35 (0.13) | 1.43 (0.13) |
| **IIIb** | 26.5 (0.08) | 26.4 (0.08) | 1.31 (0.10) | 1.30 (0.10) |
| **IV and V** | 26.6 (0.11) | 26.5 (0.10) | 1.37 (0.12) | 1.42 (0.12) |
| **P for trend** | <0.001 | <0.001 | 0.109 | <0.001 |
| All values are means and standard errors unless otherwise stated  * Adjusted for sex, age and smoking  ^†^ Adjusted for sex, age, baseline BMI, smoking and follow up time | | | | |

| **Table S2: Association between social class, baseline BMI, and weight change from baseline to follow up, for 8,196 women in the EPIC-Norfolk cohort** | | | | |
| --- | --- | --- | --- | --- |
| **Social Class** | **Baseline BMI** | | **Weight change over follow up (kg)** | |
|  | **Unadjusted** | **Adjusted*** | **Unadjusted** | **Adjusted**^†^ |
| I | 25.2 (0.17) | 25.3 (0.17) | 0.99 (0.17) | 0.90 (0.17) |
| **II** | 25.6 (0.07) | 25.7 (0.07) | 1.33 (0.08) | 1.26 (0.08) |
| **IIIa** | 25.7 (0.10) | 25.7 (0.10) | 1.26 (0.10) | 1.31 (0.10) |
| **IIIb** | 26.2 (0.10) | 26.3 (0.10) | 1.42 (0.10) | 1.36 (0.10) |
| **IV and V** | 26.7 (0.11) | 26.7 (0.11) | 1.45 (0.12) | 1.48 (0.12) |
| **P for trend** | <0.001 | <0.001 | 0.072 | <0.001 |
| All values are means and standard errors unless otherwise stated  * Adjusted for sex, age and smoking  ^†^ Adjusted for sex, age, baseline BMI, smoking and follow up time | | | | |

| **Table S3: Association between social class and risk of gaining more than 2.5 kg over the follow up period for 6,423 men in the EPIC-Norfolk cohort** | | | | |
| --- | --- | --- | --- | --- |
| **Social Class** | **Weight stable**  **gain ≤2.5kg N (%)** | **Weight gain**  **>2.5kg N (%)** | **Unadjusted odds ratio (95% CI)** | **Adjusted odds ratio^*^**  **(95% CI)** |
| **I** | 366 (9) | 160 (7) | 1 | 1 |
| **II** | 1763 (41) | 892 (41) | 1.16 (0.95 1.42) | 1.12 (0.91 1.38) |
| IIIa | 524 (12) | 294 (14) | 1.28 (1.02 1.62) | 1.28 (1.01 1.63) |
| **IIIb** | 976 (23) | 483 (22) | 1.13 (0.91 1.40) | 1.06 (0.85 1.31) |
| **IV and V** | 620 (15) | 345 (16) | 1.27 (1.01 1.60) | 1.23 (0.97 1.55) |
| **P for trend** |  |  | 0.148 | <0.001 |
| * Adjusted for sex, age, baseline BMI, smoking and follow up time | | | | |

| **Table S4: Association between social class and risk of gaining more than 2.5 kg over the follow up period for 8,196 women in the EPIC-Norfolk cohort** | | | | |
| --- | --- | --- | --- | --- |
| **Social Class** | **Weight stable**  **gain ≤2.5kg N (%)** | **Weight gain**  **>2.5kg N (%)** | **Unadjusted odds ratio (95% CI)** | **Adjusted odds ratio^*^**  **(95% CI)** |
| **I** | 394 (7) | 180 (6) | 1 | 1 |
| **II** | 1997 (38) | 1055 (37) | 1.16 (0.95 1.40) | 1.14 (0.94 1.38) |
| IIIa | 1066 (20) | 553 (19) | 1.14 (0.93 1.39) | 1.18 (0.96 1.45) |
| **IIIb** | 1038 (20) | 619 (21) | 1.31 (1.07 1.60) | 1.24 (1.01 1.53) |
| **IV and V** | 811(15) | 483 (17) | 1.30 (1.06 1.61) | 1.27 (1.02 1.56) |
| **P for trend** |  |  | 0.004 | <0.001 |
| * Adjusted for sex, age, baseline BMI, smoking and follow up time | | | | |
