# Supplementary material for: Socioeconomic position and risk of short-term weight gain: Prospective study of 14,619 middle-aged men and women
Source: BMC Public Health. 2008 Apr 9;8:112. doi: 10.1186/1471-2458-8-112 (PMC2323377; doi:10.1186/1471-2458-8-112)
Supplement: Additional file 2 — Statistical analyses with full diet and physical activity data. The data provided represent the statistical analysis for only individuals with full diet and physical activity data available. [file 1471-2458-8-112-S2.doc]

**Analyses for individuals with full diet and physical activity data**

| **Table S5: Association between social class and weight change from baseline to follow up only in individuals with full diet and physical activity data N = 12,760** | | | | |
| --- | --- | --- | --- | --- |
| **Social Class** | **N** | **Weight change over follow up (kg)** | | |
|  |  | **Unadjusted** | **Adjusted*** | **Adjusted*** **+ Mediators**^†^ |
| I | 958 | 0.90 (0.13) | 0.86 (0.13) | 0.85 (0.13) |
| **II** | 5010 | 1.29 (0.06) | 1.26 (0.06) | 1.25 (0.06) |
| **IIIa** | 2146 | 1.28 (0.08) | 1.35 (0.08) | 1.34 (0.08) |
| **IIIb** | 2725 | 1.41 (0.08) | 1.39 (0.07) | 1.41 (0.07) |
| **IV and V** | 1921 | 1.48 (0.09) | 1.52 (0.09) | 1.54 (0.09) |
| **P for trend** |  | <0.001 | <0.001 | <0.001 |
| All values are means and standard errors unless otherwise stated  * Adjusted for sex, age, baseline BMI, smoking and follow up time  ^†^ Mediators physical activity, energy intake, plasma vitamin C level | | | | |

| **Table S6: Association between social class and risk of gaining more than 2.5 kg over the follow up period only in individuals with full diet and physical activity data N = 12,760** | | | | | |
| --- | --- | --- | --- | --- | --- |
| **Social Class** | **Weight stable**  **gain ≤2.5kg N (%)** | **Weight gain**  **>2.5kg N (%)** | **Unadjusted odds ratio**  **(95% CI)** | **Adjusted odds ratio^*^**  **(95% CI)** | **Adjusted^*^ + Mediators**^†^ **odds ratio**  **(95% CI)** |
| **I** | 670 (8) | 288 (7) | 1 | 1 | 1 |
| **II** | 3323 (40) | 1687 (38) | 1.18 (1.02 1.37) | 1.16 (1.00 1.35) | 1.16 (1.00 1.35) |
| IIIa | 1401 (17) | 745 (17) | 1.24 (1.05 1.46) | 1.26 (1.07 1.49) | 1.28 (1.08 1.51) |
| **IIIb** | 1767 (21) | 958 (22) | 1.26 (1.08 1.48) | 1.20 (1.02 1.41) | 1.21 (1.03 1.42) |
| **IV and V** | 1215 (15) | 706 (16) | 1.35 (1.14 1.60) | 1.31 (1.11 1.56) | 1.33 (1.12 1.57) |
| **P for trend** |  |  | <0.001 | <0.001 | <0.001 |
| * Adjusted for sex, age, baseline BMI, smoking and follow up time  ^†^ Mediators physical activity, energy intake, plasma vitamin C level | | | | | |
